# Investigation of promoter regions, motifs, and CpG islands in the regulation of gene expression in *Trametes hirsuta* strain 072

**DOI:** 10.1186/s43141-021-00261-9

**Published:** 2021-10-18

**Authors:** Dinku Senbeta, Mulugeta Kebede

**Affiliations:** 1 Department of Applied Biology, School of Applied Natural Science, Adama Science and Technology University, P.O. Box 1888, Adama, Ethiopia; 2 Department of Biology, College of Natural & Computational Science, Adigrat University, P.O. Box 50, Adigrat, Ethiopia

**Keywords:** *Trametes hirsuta* strain 072, Promoter region, Motif, CpG island

## Abstract

**Background:**

In silico analysis of transcription start sites, promoter regions, transcription factors and their binding sites, and CpG islands for the *Trametes hirsuta* strain 072 genome were performed to understand the regulation mechanisms of gene expression and its genetic variations in the genomes. Therefore, a computational survey was carried out for the *Trametes hirsuta* strain 072 genome with the open reading frames from the National Center for Biotechnology Information database. Seventeen functional sequences were used to analyze promoter regions and their regulatory elements.

**Result:**

The present study revealed that 94% of *Trametes hirsuta* strain 072 genes contained more than two TSSs. Among these identified TSSs, a TSS with the highest predictive score was considered to determine a promoter region of the genes. Moreover, a total of five common candidate motifs such as MotI, MotII, MotIII, MotIV, and MotV were identified. Among these motifs, motif IV was investigated as the common promoter motif for 41.17% of genes that serve as binding sites for transcription factors (TFs) involved in the expression regulation of *Trametes hirsuta* strain 072 genes. Motif IV was also compared to registered motifs in publically available databases to see if they are similar to known regulatory motifs for TF using TOMTOM web server. Hence, it was revealed that MotIV might serve as the binding site mainly for the leucine zipper TF gene family to regulate a gene expression of *Trametes hirsuta* strain 072. Regarding CpG island determination, it was concluded that there is no CpG island in both promoter and gene body regions of the *Trametes hirsuta* strain 072 genome.

**Conclusions:**

This study provides a better insight into further molecular characterization which aimed to efficiently exploit a white rot fungus, *Trametes hirsuta* strain 072, for several biotechnological applications aimed to revitalize a severely contaminated environment.

## Background

White rot fungi (WRF) are crucial components of several terrestrial ecosystems [1, 2] which are fundamentally important in carbon balance, soil formation, forest regeneration, and support the biodiversity of our planet [3, 4]. They can degrade lignocellulose efficiently and make them an attractive target for several biotechnological applications, such as biofuel production, bio pulping industry, and bioremediation technology [5, 6]. Moreover, lignin is a major component of lignocellulosic biomass [7] and is mainly responsible for its strength [8–10] and used for industrial production of aromatics and novel plastics, or as a source of green energy [11].

White rot fungi are an excellent microorganism in degrading lignin and a wide range of complex organopollutants [12]. This ability is mainly associated with non-specific extracellular ligninolytic enzymes such as laccase (Lac), lignin peroxidase (LiP), manganese peroxidase (MnP), and several other peroxidases such as versatile peroxidase (VP) and humic acid peroxidases (HuP) [13, 14]. Of particular interest, MnP is the heme-containing glycoprotein that WRF mainly produces. Besides being used in the conversion of lignin and lignocelluloses, MnP has great application potential in the field of environmental biotechnology and degradation of recalcitrant organopollutants that are highly harmful to human health [15–18].

Several studies reported that *Phanerochaete chrysosporium* had been the most intensively studied WRF as a source of extracellular ligninolytic enzymes. However, the production of these enzymes particularly peroxidases with this fungus in stirred bioreactors proved to be demanding [17, 18]. Screening of WRF pointed out strains from other genera such as *Trametes*, *Pleurotus*, *Bjerkandera*, *Cerrena*, and *Echinodontium* as a suitable source of ligninolytic enzymes [19–21]. Furthermore, [22] revealed that *Trametes hirsuta* potentially produces a wide spectrum of laccase isozymes of high redox potential, an efficient lignin degrader.

Over the past few years, the use of WRF for bioremediation purposes has gained interest in the scientific community [23]. Fungal extracellular lignin modifying enzymes have been reported to be particularly efficient in removing several recalcitrant environmental wastes [24]. White rot fungi secrete an enzyme of interest in the treatment of multiple trace organic contaminants in many compartments of the environment. Thus, biotechnological approaches are explored as an eco-friendly alternative that exploits the ability of various microorganisms, specifically white rot fungi and their enzyme products, to revitalize the contaminated environment.

Analysis of promoter regions, transcription start sites (TSSs), and motifs are fundamental to understanding gene expression regulation mechanisms and association with genetic variations in the promoter regions of genomes [25, 26]. Apart from transcription factors, CpG islands are also important regulatory elements in the promoter regions of the genome and they are considered gene markers because they play important roles in gene regulation through epigenetic changes [27].

Motifs are short DNA sequences bound by one or more DNA-binding proteins or protein complexes [28]. They are often associated with specialized proteins known as transcription factors and are thus linked to transcriptional regulation [29]. The common promoter motif is the key signature for a family of co-regulated genes and is usually present in the regions where complex protein interactions occur [30]. It is also reported that genes having similar expression patterns contain common motifs in their promoter regions [31].

Moreover, to our best of knowledge, there is no study reported regarding in silico analysis of genes vital in gene expression and association with genetic variation of *Tremetes hirsuta* strain 072 in Ethiopia. Therefore, this study was intended to identify regulatory elements such as promoter regions, CpG islands, transcription factors (TFs), and their corresponding binding sites (TFBSs) involved in the regulation of gene expression mechanisms. This study provides baseline information and additional insights, which were aimed to be used efficiently for several biotechnological applications and further detail molecular characterization of the *Trametes hirsuta* strain 072 genome.

## Methods

### Determination of transcription start sites (TSSs) and promoter regions

A functional genome sequence of *Trametes hirsuta* strain 072 was taken from the NCBI genome browser (https://www.ncbi.nlm.nih.gov/gene). A total of seventeen gene coding sequences starting with the ATG codon were identified and used in this analysis. To determine their respective transcription start sites (TSSs), 1-kb sequences upstream of the start codon were excised from each gene [32]. All the TSSs of each functional gene were searched within this region by using a Neural Network Promoter Prediction (NNPP version 2.2) toolset with the minimum standard predictive score (between 0 and 1) cutoff value of 0.8 [33]. This tool helps locate the possible TSSs within the sequences upstream of the start codon where the RNA polymerases start their activity and transcription process. The Neural Network Promoter Prediction tool has the ability to recognize precisely the position of a TSS for a given gene. For those regions containing more than one TSS, the highest value of the prediction score was considered to be a trustable and accurate prediction. According to a previously reported study, a promoter sequence was defined as a 1-kb region upstream of each TSS [34, 35].

### Identification of common candidate motifs and transcription factors

Identified promoter sequences were analyzed using the MEME version 5.0.1 searches, via the web server hosted by the National Biomedical Computation Resource (http://meme.nbcr.net) [36] to look for common candidate motifs that serve as binding sites of transcription factors that regulate the expression of genes. The MEME suite software searches for statistically significant candidate motifs in the input sequence set. The MEME output was presented in the form of XML and shows the candidate motifs as local multiple alignments of the input promoter sequences. Briefly, the MEME toolset discovers novel, ungapped motifs (recurring, fixed-length patterns) in sequences submitted in it.

A motif is an approximate sequence pattern that repeatedly occurs in a group of related sequences. MEME represents motifs as position-dependent letter probability matrices that describe the probability of each possible letter at each position in the pattern. MEME takes as input a group of sequences and outputs as many motifs as requested. MEME uses statistical modeling techniques to automatically choose the best width, number of occurrences, and description for each motif [36]. Buttons on the MEME HTML output allow one or all candidate motifs to be forwarded for further analysis to better characterize the identified candidate motifs by other web-based programs, TOMTOM. The TOMTOM [37] web server was used to search for sequences matching the identified motif for its respective TF. The output of TOMTOM includes LOGOS representing the alignment of the candidate motif and TF with the *p*-value and *q*-value (a measure of false discovery rate) of the match and links back to the parent transcription database for more detailed information about it [38].

### Investigation for CpG islands

Two algorithms were used to search for CpG islands. First, the stringent search criteria, the Takai and Jones’ algorithm: GC content ≥ 55%, ObsCpG/ExpCpG ≥ 0.65, and length ≥ 500 bp were used [39]. For this purpose, the CpG island searcher program (CpGi130) is available at web link http://dbcat.cgm.ntu.edu.tw/ was used. Secondly, the CLC Genomics Workbench ver. 3.6.5 (http://clcbio.com, CLC bio, Aarhus, Denmark) was used for searching the restriction enzyme *MspI* cutting sites (fragment sizes between 40 and 220 bps). Searching for *MspI* cutting sites is relevant for the detection of CGIs because studies using whole-genome CpG island libraries prepared for different species revealed that, CpG islands are not randomly distributed but are concentrated in particular regions because CpG-rich regions are achieved by isolation of short fragments after *MspI* digestion that recognizes CCGG sites [40].

## Results

### Identification of TSSs and promoter regions for each gene of *Trametes hirsuta* strain 072

Gene coding sequences were used in this analysis. The TSSs were identified for each functional gene by excising 1-kb sequences upstream of the start codon, indicating that regulatory elements of the core promoter may lie up within this region. In this study, only cox2/36279600 genes have a single TSS, whereas 98% of genes contained more than two TSSs. Moreover, only the nad4/36279629 gene has a maximum of nine TSSs. Regarding predictive score, 56% of the cases were greater than or equal to 0.90 (Table 1).

**Table 1:** Identified TSSs and predictive score value for *Trametes hirsuta* strain 072

Name/gene ID	Name of corresponding promoter region	No. of TSSs	Predictive score (at cutoff value 0.8)	Distance from start codon (ATG)
atp9/36279644	Pro-36279644	2	0.98, 0.97	-74, -1221
nad3/36279640	Pro-36279640	2	0.94, 0.82	-801, -1309
nad2/36279639	Pro-36279639	3	1.00, 0.90, 0.88	-796, -1390, -1456
nad4/36279629	Pro-36279629	9	0.99, 0.96, 0.91,0.84, 0.98, 0.86,0.89, 0.92, 0.82	-3402, -3481, -3913, -4008, 617, -528, -2963, -3096, -4847
C7V85mgp08/36279625	Pro-36279625	5	0.99, 0.56, 0.86, 0.87, 0.81	-790, -1077, -1120, -953, -257
cox1/36279622	Pro-36279622	2	0.91, 0.85	-810, -310
Cob/36279616	Pro-36279616	3	0.98, 0.96, 0.92	-759, -737, -499,
nad1/36279614	Pro-36279614	6	0.99, 0.83, 0.95, 0.96, 0.94, 0.86	-1644, -1779, -2357, -1627, -1381, -607
atp8/36279610	Pro-36279610	5	0.99, 0.96, 0.91, 0.98, 0.98	-733, -934, -1131, -1394, -254
nad5/36279609	Pro-36279609	4	0.99, 0.86, 0.85, 0.94	-2760, -3044, -3261, -3739
nad4L/36279608	Pro-36279608	4	0.99, 0.86, 0.85, 0.94	-2493, -2777, -2994, -3472
cox3/36279607	Pro-36279607	5	0.99, 0.86, 0.85, 0.94, 0.91	-1324, -1608, -1825, -2303, -2940
C7V85mgp19/36279606	Pro-36279606	8	0.91, 1.00, 0.89, 0.94, 0.96,1.00, 0.83, 0.86	-443, -1055, -1526, -2204, -2619, -2698, -2706, -2776
atp6/36279604	Pro-36279604	6	0.84, 0.84, 1.00, 0.82, 0.85, 0.89	-1396, -2512, -2655, -2921, -3051, -3576
nad6/36279602	Pro-36279602	5	1.00, 0.84, 0.84, 0.82, 0.85	-413, -556, -822, -952, -1477
s3/36279601	Pro-36279601	3	0.99, 0.99, 0.84	-26, -1132, -2174
cox2/36279600	Pro-36279600	1	0.99	-980

### Common candidate motifs and associated TFs in the promoter regions of *Trametes hirsuta* strain 072

**Fig. 1:**
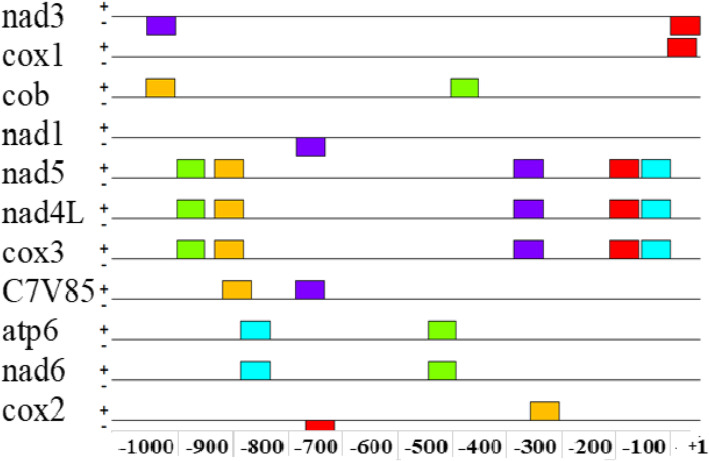
Positions of candidate motifs in different promoter sequences of *Trametes hirsuta* strain 072 relative to TSSs (from + 1 TSS to the upstream − 1000 kb)

**Fig. 2:**
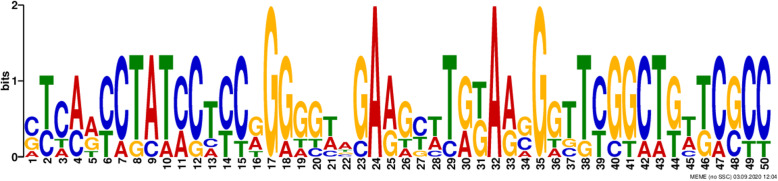
Sequence logos for identified common promoter motif, motif IV of *Trametes hirsuta* strain 072 using MEME Suite

Identified promoter sequences for *Trametes hirsuta* strain 072 were analyzed using the MEME searches (http://meme.nbcr.net) to look for common candidate motifs that serve as binding sites of TFs that regulate the expression of genes. MEME searches for statistically significant candidate motifs in the input sequence set. In the current study, five candidate motifs shared by most input promoter sequences were investigated (Table 2). The location and distribution of these candidate motifs in the promoter regions are concentrated between − 700 bp and 800 bp relative to the TSSs (Fig. 1). It was also noticed that twenty-five motifs were distributed on the positive strand, whereas four motifs were distributed on a negative strand (Fig. 1). In the present study, Motif IV was revealed as the binding site for TFs involved in the expression regulation of these genes (Table 2). Moreover, the sequence logo for motif IV is also presented in Fig. 2.

**Table 2:** Identified common candidate motifs in *Trametes hirsuta* strain 072 gene promoter regions

Discovered candidate motif	Number (%) of promoters containing each one of the motifs	*E*-value	Motif width	Total no. of binding sites
MotI	6(35.29%)	6.9e−027	50	6
MotII	5(29.41%)	1.5e−0.25	50	5
MotIII	6(35.29%)	3.0e−0.19	47	6
MotIV	7(41.17%)	1.1e−0.18	50	7
MotV	6(35.29%)	4.3e−0.20	49	6

The TOMTOM web server [37] was further used to get more information on the motif IV promoter genes. Motif IV was compared to already documented and publically available databases to see if they are similar to known regulatory motifs for TFs. Accordingly, motif IV matched with four known motifs found in databases. Among four identified matched motifs, only three TF families were considered in the study, and a left query motif was non-transcription factor families (Table 3). Moreover, it was also revealed that motif IV serves as binding sites for many transcription factor (TFs) families. Based on their statistical significance values, leucine zipper transcription factor families (6.17e−04) were involved in the regulatory mechanism of *Trametes hirsuta* strain 072 genes, which used to enhance a transcription process.

**Table 3:** Identified transcription factors which could bind to motif IV

Classification of TF families	Candidate transcription factors	Gene	Statistical significance
Leucine zipper	Leucine type	OPI1	6.17e−04
Myb/SANT domain factors	Tryptophan cluster factors	RAX3	2.11e−03
Ets-related factors	Tryptophan cluster factors	EWSRI-FLI1	5.35e−03

### Determination of CpG islands in promoter regions of *Trametes hirsuta* genes

CpG islands were also investigated to determine regulatory elements in *Trametes hirsuta* in both promoter and gene body regions using two algorithms. Initially, in silico analysis using Takai and Jone’s algorithm [39] found no CpG islands in both promoter regions in *Trametes hirsuta* strain 072 (Table 4). Similarly, a second approach to explore the presence of CpG islands in silico digestion was performed using the restriction enzyme *MspI*, which revealed poor CpG islands in both promoter and gene body regions (Table 5). CpG islands from only the atp8 gene contained a single fragment size of 141 bp in its promoter region. However, there were no CpG island–specific sequences in the other ten promoter sequences of *Trametes hirsuta* strain 072 genes. Moreover, CpG islands were only recognized in the Cox1 gene with a fragment size of 17bp and 114bp (Table 5), which is poor CpG islands in this species.

**Table 4:**
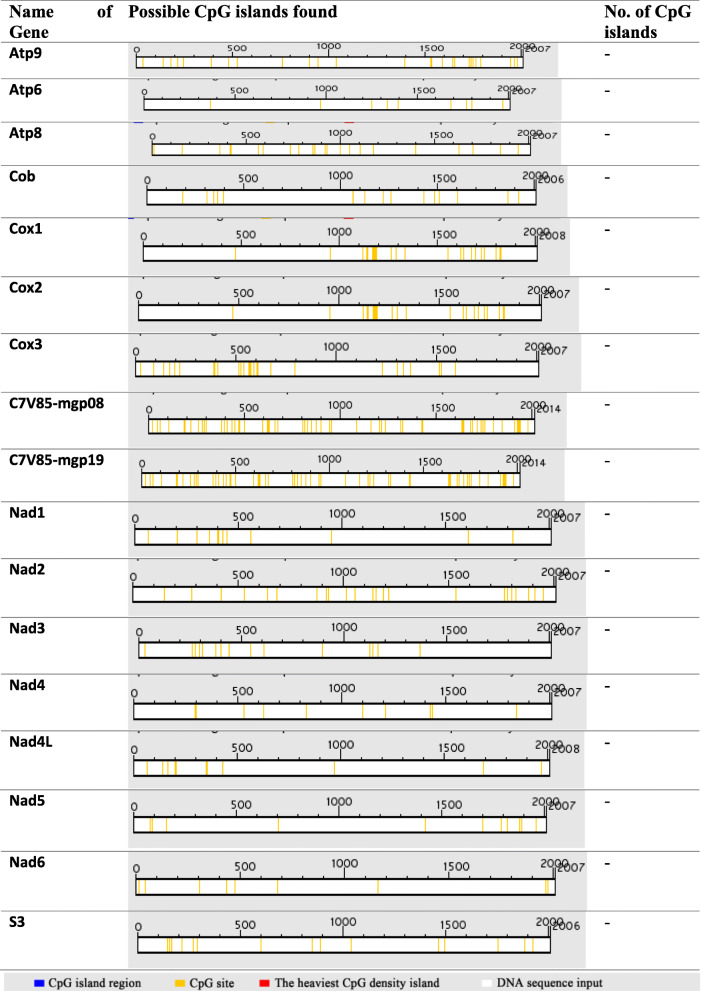
Possible CpG islands in promoter regions using Takai and Jones’ algorithm

**Table 5:** Determination of *MspI* cutting sites and fragment sizes for *Trametes hirsuta* strain 072 promoter regions

Region	No. of corresponding promoter gene of *T.hirsuta*	Nucleotide positions of *MspI* sites	Fragment size (40 and 220 bps)
Promoter region	Prom-atp9	Single cut (at383	–
	Prom-atp6	No cut	–
	Prom-atp8	Multiple cut (at 586,727,1043,1393)	141
	Prom-C7V85-mgp08	Double cut (at 1189,1548)	–
	Prom-C7V85-mgp19	Single cut (at 1788)	–
	Prom-cob	Single cut (at 1118)	–
	Prom-cox1	Double cut(at1257,1793)	–
	Prom-cox2	Double cut (at 1257,1793)	–
	Prom-cox3	Single cut (at 534)	–
	Prom-nad1	No cut	–
	Prom-nad2	No cut	–
	Prom-nad3	Single cut (at 605)	–
	Prom-nad4	Single cut(at 290)	–
	Prom-nad4l	Single cut(at 1962)	–
	Prom-nad5	Single cut (at 1695)	–
	Prom-nad6	Single cut (at 676)	–
	Prom-s3	No cut	–
Gene body region	Atp9	Single cut (at 115)	–
	Atp6	No cut	––
	Atp8	No cut	–
	Cob	No cut	–
	Cox1	Multiple cut (1056, 1343, 1782, 1799, 1913, 3286, 3645, 4313, 6145, 7526	17,114
	Cox2	No cut	–
	Cox3	No cut	–
	C7V85-mgp08	Single cut (at 216)	–
	C7V85-mgp19	No cut	–
	Nad1	Single cut (948)	–
	Nad2	Single cut (at 112)	–
	Nad3	No cut	–
	Nad4	No cut	–
	Nad4L	No cut	–
	Nad5	No cut	–
	Nad6	No cut	–
	S3	Single cut (at 127)	–

## Discussion

Transcription start site (TSS) and promoter region identifications are the first steps to understanding gene expression regulatory mechanisms and association with genetic variations in the regions [25, 26, 41]. In this study, the TSSs were first identified for each of the seventeen functional genes of *Trametes hirsuta* strain 072. The prediction is more reliable for genes containing more than one TSS; TSS of the highest prediction score was considered and identified. In silico analysis showed that only cox2/36279600 genes have a single TSS, whereas 98% of genes contained more than two TSSs. Moreover, only the nad4/36279629 gene has a maximum of nine TSSs (Table 1). The current finding is in contrast to a study reported by Yirgu and Kebede [34] in which 37.9% have more than one TSS, whereas 62.1% had only one TSS. A possible reason for the discrepancy of results between the studies might be related to differences in the genome size of the studied organisms. Moreover, Dinka and Minh [42] reported that 70% of the pig V1R genes have more than one TSS, which is in line with the present study where 94% of the *Trametes hirsuta* strain 072 genes have more than one TSS.

A candidate motif that is common to most of *Trametes hirsuta* strain 072 gene promoter sequences was identified. In the current study, five candidate motifs shared by most input promoter sequences were investigated (Table 2). The location and distribution of these candidate motifs in promoter regions are concentrated between − 700 bp and − 800 bp relative to the TSSs (Fig. 1). This finding disagrees with the study reported by Samuel and Dinka [26], which revealed that 73.9% of the TSSs were found within − 500 bp relative to the translation start codon. Moreover, Chen et al. [43] also indicated that multiple TSSs for the human GnT-II were concentrated between − 440 to − 489 bp relative to the ATG translation start codon. MEME toolset generated a common candidate motif for eleven *Trametes hirsuta* strain 072 genes promoter sequences with a total of seventeen input sequences. It was also noticed that twenty-five motifs were distributed on the positive strand, whereas four motifs were distributed on a negative strand (Fig. 1). Yirgu and Kebede [34] showed that higher distributions of motifs were found in positive strands (96) than negative strands (81) in the *H. seropedicae* ACP92s gene. Motifs shared by most promoter regions were considered candidate motifs that are functionally involved in the gene regulation mechanisms. In the present study, motif IV was revealed as the common promoter motif for 41.17% of genes that serve as binding sites for TFs involved in the expression regulation of these genes (Table 2). Moreover, the sequence logo for motif IV is also presented in Fig. 2.

The TOMTOM web server [37] was further used to get more information on the motif IV promoter genes. Motif IV was compared to already documented and publically available databases to see if they are similar to known regulatory motifs for TFs. Accordingly, motif IV matched with four known motifs found in databases. Among four identified matched motif, only three TF families were considered in the study and a left query motif was non-transcription factor families (Table 3). Moreover, it was also revealed that motif IV serves as binding sites for many transcription factor (TF) families. On the basis of their statistical significance values, leucine zipper transcription factor families (6.17e−04) were involved in the regulatory mechanism of *Trametes hirsuta* strain 072 genes. Loewen et al. [44] stated that transcriptional repressor OPI1 had a transcription co-repressor activity as molecular functions.

CpG islands were also investigated to determine regulatory elements in the *Trametes hirsuta* in both promoter and gene body regions using two algorithms. Initially, in silico analysis using Takai and Jones’ algorithm [39] found no CpG islands in promoter regions in *Trametes hirsuta* strain 072 (Table 4). This result is inconsistent with the study conducted by Yirgu and Kebede [34], which revealed there was one possible CpG island in most of the genes both in promoter and gene body regions. Moreover, this study is consistent with studies that reported poor CpG islands in both promoter and gene body regions [26, 42, 45]. Similarly, a second approach to explore the presence of CpG island in silico digestion was performed using restriction enzyme *MspI*, which revealed poor CpG islands in both promoter and gene body regions (Table 5). CpG islands from only the atp8 gene contain a single fragment size of 141 bp in its promoter region. However, there were no CpG island–specific sequences in the other ten promoter sequences of *Trametes hirsuta* strain 072 genes. Moreover, CpG islands were only recognized in the Cox1 gene with a fragment size of 17 bp and 114 bp (Table 5) and concluded as poor in CpG islands in this species. Consequently, the present result disagreed with the study of Yirgu and Kebede [34], which concluded that *H. seropedicae* ACP92 genes were rich in CpG islands and were consistent with the studies of [26, 42, 45] summarized as poor in CpG islands in different eukaryotic organisms.

## Conclusions

In silico analysis of transcription start sites (TSSs), promoter regions, transcription factors and their binding sites, and CpG islands for the *Trametes hirsuta* strain 072 genome was performed to better understand the regulation mechanisms of gene expression and its genetic variations in the genomes. This study revealed that 94% of *Trametes hirsuta* strain 072 genes contained more than two TSSs. Therefore, among identified TSSs, a TSS with the highest predictive score was considered to determine the genes’ promoter regions. Moreover, five common candidate motifs such as MotI, MotII, MotIII, MotIV, and MotV were identified. Among these motifs, motif IV was investigated as the common promoter motif for 41.17% of genes that serve as binding sites for transcription factors (TFs) involved in the gene expression and regulation mechanisms of *Trametes hirsuta* strain 072 genes.

Regarding CpG island determination, it was concluded that there are no CpG islands in both promoter and gene body regions of the *Trametes hirsuta* strain 072 genes but could still be expressed when they are methylated. In silico analysis is pivotal in identifying gene promoter regions and other regulatory elements relevant to predict gene expression mechanisms in organisms. Additionally, this study provides better insights into molecular characterization, which aimed to efficiently exploit white rot fungi, *Trametes hirsuta* strain 072, for several biotechnological applications and revitalize a contaminated environment.

## Data Availability

The research data analyzed in the current study is available and taken from the assembly of the NCBI genome assembly browser of *Trametes hirsuta* strain 072.

## Abbreviations

TSS: Transcription start site
TFs: Transcription Factors
MEME: Multiple Em for motif elicitation
NCBI: National Center for Biotechnology Information
bp: Base pair
NNPP: Neural Network Promoter Prediction
WRF: White rot fungi
Mot: Motif
